# Supplementary material for: Characterisation of Extracts Obtained from Unripe Grapes and Evaluation of Their Potential Protective Effects against Oxidation of Wine Colour in Comparison with Different Oenological Products
Source: Foods. 2021 Jun 28;10(7):1499. doi: 10.3390/foods10071499 (PMC8305229; doi:10.3390/foods10071499)
Supplement: Supplementary file 1 [file foods-10-01499-s001.zip › foods-1259996-supplementary.pdf]

Table S1: Pearson's correlation coefficients of antioxidant activity determined by the DPPH spectrophotometric assay and chemical parameters of wines.

Variable	AT	VA	pH	Alcohol	Free $_{SO2}$	Total SO_2	TP (Folin)	Abs 420	Abs 520	Abs 620	CI	Hue	AA
AT	**1**	-0.037	**-0.554**	**0.886**	**-0.663**	**-0.624**	**0.829**	**0.768**	**0.770**	0.156	**0.712**	-0.025	**0.707**
VA	-0.037	**1**	0.169	-0.032	-0.355	-0.241	-0.203	0.133	0.079	-0.072	0.081	0.135	-0.105
pH	**-0.554**	0.169	**1**	**-0.531**	-0.002	-0.019	**-0.811**	**-0.374**	**-0.370**	0.030	-0.327	0.013	**-0.544**
Alcohol	**0.886**	-0.032	**-0.531**	**1**	**-0.648**	**-0.674**	**0.783**	**0.605**	**0.530**	-0.065	**0.493**	0.243	**0.717**
Free SO_2	**-0.663**	-0.355	-0.002	**-0.648**	**1**	**0.945**	-0.349	**-0.735**	**-0.663**	-0.359	**-0.680**	-0.200	**-0.676**
Total SO_2	**-0.624**	-0.241	-0.019	**-0.674**	**0.945**	**1**	-0.338	**-0.736**	**-0.642**	**-0.393**	**-0.676**	-0.286	**-0.691**
TP (Folin)	**0.829**	-0.203	**-0.811**	**0.783**	-0.349	-0.338	**1**	**0.709**	**0.678**	0.200	**0.650**	0.092	**0.735**
Abs 420	**0.768**	0.133	**-0.374**	**0.605**	**-0.735**	**-0.736**	**0.709**	**1**	**0.955**	**0.688**	**0.984**	0.112	**0.799**
Abs 520	**0.770**	0.079	**-0.370**	**0.530**	**-0.663**	**-0.642**	**0.678**	**0.955**	**1**	**0.643**	**0.980**	-0.186	**0.683**
Abs 620	0.156	-0.072	0.030	-0.065	-0.359	**-0.393**	0.200	**0.688**	**0.643**	**1**	**0.757**	0.114	**0.525**
CI	**0.712**	0.081	-0.327	**0.493**	**-0.680**	**-0.676**	**0.650**	**0.984**	**0.980**	**0.757**	**1**	-0.026	**0.743**
Hue	-0.025	0.135	0.013	0.243	-0.200	-0.286	0.092	0.112	-0.186	0.114	-0.026	**1**	0.338
AA	**0.707**	-0.105	**-0.544**	**0.717**	**-0.676**	**-0.691**	**0.735**	**0.799**	**0.683**	**0.525**	**0.743**	0.338	**1**

Total acidity (TA); volatile acidity (VA); total phenol (TP); antioxidant activity (AA); clour intensity (CI); hue of colour (H).
